# Radiomics as a tool for prognostic prediction in transarterial chemoembolization for hepatocellular carcinoma: a systematic review and meta-analysis

**DOI:** 10.1007/s11547-024-01840-9

**Published:** 2024-07-26

**Authors:** Kaige Deng, Tong Chen, Zijian Leng, Fan Yang, Tao Lu, Jingying Cao, Weixuan Pan, Yongchang Zheng

**Affiliations:** 1 Department of Liver Surgery, Peking Union Medical College Hospital, Chinese Academy of Medical Sciences, Beijing, 100730 China; 2 Department of Medical Oncology, Cancer Hospital, Chinese Academy of Medical Sciences, Beijing, 100021 China; 3 Department of Pathology, Peking Union Medical College Hospital, Chinese Academy of Medical Sciences, Beijing, 100730 China; 4 Zunyi Medical University, Zunyi, Guizhou 563000 China

**Keywords:** Hepatocellular carcinoma (HCC), Transarterial chemoembolization (TACE), Prognosis, Radiomics, Systematic review, Meta-analysis

## Abstract

**Introduction:**

Transarterial chemoembolization (TACE) is one of the predominant locoregional therapeutic modalities for addressing hepatocellular carcinoma (HCC). However, achieving precise prognostic predictions and effective patient selection remains a challenging pursuit. The primary objective of this systematic review and meta-analysis is to evaluate the efficacy of radiomics in forecasting the prognosis associated with TACE treatment.

**Methods:**

A comprehensive exploration of pertinent original studies was undertaken, encompassing databases of PubMed, Web of Science and Embase. The studies' quality was meticulously evaluated employing the quality assessment of diagnostic accuracy studies 2 (QUADAS-2), the radiomics quality score (RQS) and the METhodological RadiomICs Score (METRICS). Pooled statistics, along with 95% confidence intervals (95% CI), were computed for sensitivity, specificity, positive likelihood ratio (PLR), and negative likelihood ratio (NLR). Additionally, a summary receiver operating characteristic curve (sROC) was generated. To discern potential sources of heterogeneity, meta-regression and subgroup analyses were performed.

**Results:**

The systematic review incorporated 29 studies, comprising a total of 5483 patients, with 14 studies involving 2691 patients qualifying for inclusion in the meta-analysis. The assessed studies exhibited commendable quality with regard to bias risk, with mean RQS of 12.90 ± 5.13 (35.82% ± 14.25%) and mean METRICS of 62.98% ± 14.58%. The pooled sensitivity was 0.83 (95% CI: 0.78–0.87), specificity was 0.86 (95% CI: 0.79–0.92), PLR was 6.13 (95% CI: 3.79–9.90), and NLR was 0.20 (95% CI: 0.15–0.27). The area under the sROC was 0.90 (95% CI: 0.87–0.93). Significant heterogeneity within all the included studies was observed, while meta-regression and subgroup analyses revealed homogeneous and promising findings in subgroups where principal methodological variables such as modeling algorithms, imaging modalities, and imaging phases were specified.

**Conclusion:**

Radiomics models have exhibited robust predictive capabilities concerning prognosis subsequent to TACE, thereby presenting promising prospects for clinical translation.

**Supplementary Information:**

The online version contains supplementary material available at 10.1007/s11547-024-01840-9.

## Introduction

Hepatocellular carcinoma (HCC) represents the most prevalent form of primary liver cancer, ranking sixth globally in cancer incidence and third in cancer-related mortality. In 2020, there were approximately 906,000 new cases and 830,000 deaths attributed to HCC worldwide [1]. Recognized for its highly aggressive nature, HCC is associated with a generally poor prognosis. Age-standardized 5-year survival rates exhibit regional variations, typically ranging from 5% to 30%, and falling within 10% to 19% in most countries [2]. Therapeutic advancements have contributed to an enhanced outcome for HCC patients, with observed increases of 5% to 10% in age-standardized 5-year survival rates across different regions from 1995 to 2014 [2, 3]. Current therapeutic strategies highlight optimal management through comprehensive approaches, primarily centered around surgical interventions [4–6]. Non-surgical treatments, including transarterial chemoembolization, hepatic arterial infusion chemotherapy [7], ablations [8–10], radiotherapy [11], and systemic therapies, among others, have provided opportunities to improve the prognosis, particularly for early-stage patients unsuitable for operations and those with advanced unresectable HCC.

Transarterial chemoembolization (TACE) stands as a common locoregional treatment for liver cancer. Depending on the therapeutic agents employed, it can be broadly classified into conventional TACE (cTACE), utilizing a mixture of iodized oil and chemotherapeutic drugs, and drug-eluting bead TACE (DEB–TACE) [12]. TACE is considered the preferred therapeutic modality for liver-limited diseases deemed unsuitable for surgical resection [4], especially recommended for intermediate-stage tumors according to the Barcelona clinic liver cancer (BCLC) classification [5]. Notably, TACE finds application in various scenarios, such as the management of liver cancer with localized portal vein tumor thrombosis with incomplete obstruction, bridging therapy for liver transplantation, postoperative adjuvant therapy for patients at high risk of recurrence, and conversion therapy for unresectable tumors [4–6, 13], among others. In real-world practice, TACE is commonly employed in patients with recurrent or progressed HCC and is often repeated following the initial treatment [12]. This renders the patient selection and efficacy evaluation susceptible to the influences of previous or concurrent anti-tumor treatments. Consequently, the choice and application of TACE heavily depend on the expertise and preferences of physicians, lacking well-recognized supportive informative tools.

The assessment of tumor response and long-term prognosis subsequent to TACE primarily relies on radiological criteria, such as the response evaluation criteria in solid tumors (RECIST) [14] and modified response evaluation criteria in solid tumors (mRECIST) [15, 16]. According to these criteria, the objective response rates (ORR) after TACE exhibit notable diversity across different reports, ranging broadly from 30% to 80% [17–21]. This substantial variability may be linked to factors such as distinct baseline patient characteristics, the intricacy of background therapies before TACE, and variations in TACE techniques. Consequently, predicting prognosis following TACE could enhance the process of individualized patient selection, ultimately improving clinical outcomes [13]. Researchers have explored diverse indicators for post-TACE prognosis, encompassing radiological features [22, 23], clinico-pathological factors [24], serum markers [25], and genomic alterations [26], among others. However, these predictive tools remain hindered by limitations such as insufficient predictive power, lack of validations, and the associated trauma or cost related to these tests.

First systematically defined by Lambin et al. in 2012 [27], radiomics is a technique leveraging computer programs to explore and analyze high-throughput quantitative features from medical images. Since its inception, the influence of radiomics has progressively expanded. Radiomics has primarily been applied to aid in cancer diagnosis and prognostic prediction, offering robust support for precision medicine. By transforming medical images into a high-dimensional space of features for analysis and model construction, radiomics contributes to an enhanced understanding of medical images. In response to the unmet demand for personalized TACE, many researchers have developed radiomics models to predict prognosis after TACE. These studies have analyzed data from various imaging modalities, including magnetic resonance (MR) [21, 28–36], computed tomography (CT) [20, 37–53], as well as ultrasound (US) [54], reporting promising results. Recognizing the significance of developing reliable tools for prognostic prediction in TACE, we deem it timely and crucial to summarize the predictive performance and potential for clinical translation of radiomics models in this task. Therefore, this systematic review and meta-analysis aimed to determine the value of radiomics in predicting the prognosis of TACE treatment.

## Methods

This systematic review adheres to the preferred reporting items for systematic review and meta-analysis of diagnostic test accuracy studies (PRISMA-DTA) guidelines [55] as well as the assessing the quality of systematic reviews 2 (AMSTAR 2) guidelines [56]. The protocol was prospectively registered on PROSPERO (ID: CRD42023449278).

### Literature retrieving

A computerized search was executed on PubMed, Web of Science and Embase databases to identify original studies implementing radiomics analysis of preprocedural images to predict the therapeutic outcomes of TACE. The search strategy was devised following the PICO (participants, intervention, control, outcome) principles [55] to facilitate a sensitive screening for relevant studies. The search terms encompassed four perspectives, including "hepatocellular carcinoma or liver cancer," "radiology or radiomics," "TACE," and "prognosis." The search scope included literature published up to May 16, 2023. Detailed search conditions are provided in Supplementary Table 1.

### Study inclusion

After eliminating duplicates, the remaining literature underwent a comprehensive review based on the following inclusion criteria: (1) population: patients diagnosed with HCC through clinical or pathological confirmations; (2) intervention: initial TACE treatment (without prior history of TACE treatment); (3) index test: radiomics analysis/high-throughput analysis/artificial intelligence analysis based on preprocedural medical imaging; (4) outcomes: prognosis after TACE, including tumor response, overall survival, recurrence, progression, etc. Studies were then excluded based on the following criteria: (1) studies involving non-human subjects; (2) conference abstracts, reviews, case reports, commentaries, and other non-original publications; (3) studies not published in English; (4) studies that did not report standalone radiomics models without combining clinical features; (5) studies that did not provide sufficient raw data for qualitative or quantitative review.

The identified articles underwent an initial screening based on titles and abstracts. Subsequently, a full-text review was conducted for potentially eligible articles. The literature was reviewed by two authors (KGD, FY) with over four years of experience in hepatic surgery and medical imaging analysis. Any uncertainties were resolved through consensus in an author team, including a senior author with over fifteen years of experience in the management of liver cancers and meta-analysis (YCZ). When sufficient data were still unavailable after the full-text review, attempts were made to acquire the original data by contacting the corresponding authors. Endnote (version 20, Clarivate Analytics, England) was utilized for all literature management.

### Data extraction

Predefined information was systematically extracted from the included literature according to the following categories: (1) bibliographic features, including authors, publication year, country, and study type; (2) patient characteristics, encompassing (2–1) demographic features such as sample size, age and gender; (2–2) etiological features, such as hepatitis viral infection status and liver cirrhosis; (2–3) clinical-pathological features, including pathological diagnosis and tumor staging; (3) procedural information related to TACE; (4) pre-treatment radiological examination details, covering imaging modalities, instrument parameters, and phases or sequences of the images; (5) post-treatment follow-up information, comprising follow-up endpoints, assessment criteria, follow-up intervals, and follow-up examinations; (6) methodological characteristics of radiomics analysis, involving image segmentation methods, feature extraction methods, modeling algorithms, predictive outcomes, etc.; (7) model validation methods; (8) model performance parameters, such as sensitivity, specificity, concordance index, area under the curve (AUC), number of true positive (TP), false positive (FP), true negative (TN), false negative (FN) quantities, etc.; and (9) other relevant information. Detailed data extraction strategies are presented in Supplementary Table 2.

In instances where a single study involved the construction of multiple radiomics models, the model demonstrating the highest performance was selected for data extraction. When a study presented results for both training and validation sets [57], outcomes were extracted separately from each dataset. If an article reported both pure radiomics models and combined models incorporating additional clinical features, only results from the pure radiomics models were extracted, while the supplementary clinical features were retained for study quality evaluation.

### Quality assessment

The quality of the included studies was evaluated using the quality assessment of diagnostic accuracy studies 2 (QUADAS-2) [58], the radiomics quality score (RQS) [59] criteria, and the METhodological RadiomICs Score (METRICS) [60]. QUADAS-2 was employed to assess the risk of bias across four domains: patient selection, index test, reference standard, and flow and timing within the studies. RQS is a specialized rating framework for radiomics, covering sixteen aspects such as image acquisition, feature extraction, modeling methods, model validation, and clinical applications. And the METRICS is a newly proposed assessment tool for radiomics studies, which is based on international consensus and a well-constructed framework including ranking and weighting of methodological variations (refer to Supplementary Table 3 for details).

Throughout the data extraction and quality assessment process, a systematic approach was adopted. Initially, a training phase was initiated, during which three authors (KGD, ZJL, YCZ) independently reviewed three included papers. These authors aligned their interpretations of each item in the data extraction forms and the quality rating forms, with all definitions ultimately confirmed by senior author YCZ. Subsequently, two authors (KGD, ZJL) independently conducted data extraction and quality assessment. METRICS scores were assessed using the convenient web-based tool at https://metricsscore.github.io/metrics/METRICS.html. The final electronic datasheet integrated information unanimously confirmed by both authors.

### Statistical analysis

Statistical analysis and visualization were carried out using Microsoft Office Excel 2019, RevMan 5.3.5 [61], and Stata Statistical Software 17 (StataCorp., T.X., USA) [62]. 2 × 2 diagnostic contingency tables, containing numbers of TP, FP, FN, and TN, were extracted wherever possible. Meta-analysis was conducted using the Stata midas package [63]. Pooled sensitivity, specificity, positive likelihood ratio (PLR), and negative likelihood ratio (NLR) were calculated as synthetic statistics. A PLR > 10 or within 5–10, along with an NLR < 0.1 or within 0.1–0.2, indicates high or moderate informative value, respectively. Cochrane’s *Q*-test and *I*^2^ were utilized to assess inter-study heterogeneity, with *I*^2^ > 50% or *P* < 0.1 indicating the presence of heterogeneity. The Galbraith method was employed to identify the impact of outliers. The summary receiver operating characteristic curve (sROC) was used to evaluate the overall predictive performance of different radiomics models. An AUC of 0.5–0.7, 0.7–0.9, and > 0.9 indicates low, moderate, and high predictive power, respectively [64]. Meta-regression was used to explore the relationship between major methodological factors and inter-study heterogeneity, followed by further subgroup analyses. Deek’s funnel plot and asymmetry test were applied to evaluate publication bias. Finally, the clinical application of radiomics models was assessed. The calculated average ORR in included studies was used as prior probability, and then post-test probability was calculated using Fagan plot based on the pooled PLR and NLR and Bayesian conditional probability theorem.

## Results

### Literature retrieving, selection and data extraction

**Fig. 1:**
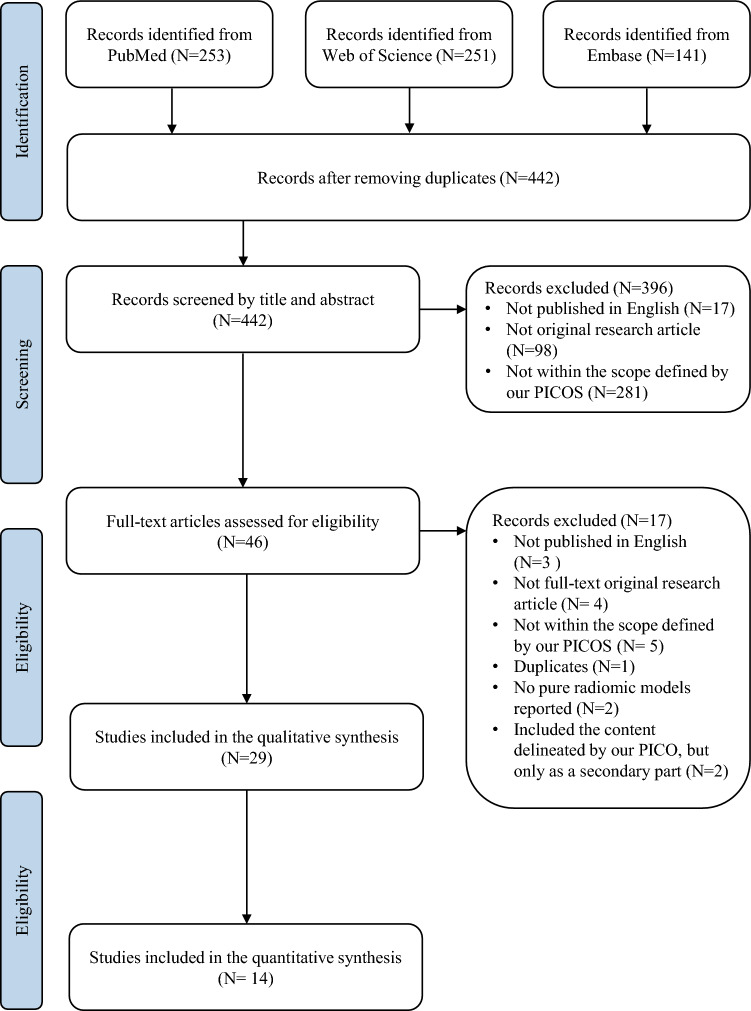
PRISMA flowchart illustrating the literature selection process in this study.

The literature retrieval process identified a total of 645 relevant articles, and 203 duplicated articles were subsequently removed. From the remaining 442 studies, scrutiny of titles and abstracts led to the exclusion of 396 articles that did not align with the language, study type, or PICO criteria specified for our review. After a thorough review of full texts, an additional 17 articles were excluded, resulting in the final inclusion of 29 articles (Fig. 1).

Summarized information for all 29 included studies is presented in Table 1, covering a total of 5483 patients. Details of the studies are presented in Supplementary Table 4, with the primary research features illustrated in Supplementary Fig. 1. The majority of the articles originated from China (24 articles, 82.8%). The included studies date back to 2016, with a progressive increase in the number of publications, peaking at 19 studies in 2021–2022. All included studies adopted a retrospective design. Most studies utilized the BCLC staging system to determine the inclusion of patients, while others used the China liver cancer staging (CNLC) system [32] or other criteria [21, 31, 40, 43, 48]. Mid-stage patients were the most extensively studied population, with 21 articles (in whole or in part) including BCLC-B stage patients [20, 28–30, 33, 37, 41, 28–30, 49, 53], followed by BCLC-A (13 articles) and BCLC-C stage (9 articles) patients.

**Table 1:** Baseline characteristics of the included studies

Year, author, nation	Imaging modality	Number of patients	Tumor staging	TACE procedure	Region of interest	Modeling algorithm	Number, phase of features	Validation datasets	Predicted endpoints
2020, Liu, China [54]	CEUS	130	BCLC-B	cTACE	3D	CNN	NA, NA	internal validation	OR
2021, Chen, China [37]	CECT	595	BCLC-B	NA	3D	LASSO	18, AP + PS	internal and external validation	OR
2021, Jin, China [38]	CECT	256	BCLC-A/B	cTACE	3D	LDA	14, PVP	external validation	EVIT
2021, Peng, China [39]	CECT	310	BCLC-B	cTACE	3D	CNN, RF	14, AP	internal validation	OR
2022, Bai, China [20]	CECT	111	BCLC-A/B	cTACE	3D	SVM	22, AP + VP	internal validation	OR
2022, Li, China [40]	CECT	48	< = 2 tumors	NA	3D	LASSO	5, early AP + mid AP + late AP + courage period	resampling validation	OR
2022, Wang, China [41]	CECT	243	BCLC-A/B	cTACE or DEB-TACE	3D	LASSO, XGBoost	6, AP + PVP	internal validation	Unsuitable for TACE
2023, Sun, China [42]	CECT	399	BCLC-B	cTACE	3D	LASSO	19, AP	internal validation	OR
2020, Sun, China [28]	CEMR	84	BCLC-B	cTACE	3D	LASSO	NA, T2WI + DWIb0_DWIb500 + ADC	internal validation	PD
2021, Kong, China [29]	CEMR	99	BCLC-B/C	cTACE or DEB-TACE	3D	LASSO	6, T2WI	internal validation	OR
2021, Zhao, China [30]	CEMR	122	BCLC-A/B/C	cTACE	3D	LASSO	6, AP + PVP + DP	internal validation	OR
2022, Cannella, Italy [31]	CEMR	51	Unifocal tumor	NA	3D	hybrid descriptive- inferential method	3, PVP	fivefold cross-validation	OR
2022, Tian, China [32]	CEMR	71	CNLC-Ib/IIa	cTACE	3D	3D-CNN, SVM-RFE	20, T2WI	fivefold cross-validation	PD
2023, Chen, China [33]	CEMR	172	BCLC-A/B/C	cTACE or DEB-TACE	3D	DNN	NA, T2WI + AP + PVP + DP	internal and external validation	OR
2016, Hinrichs, Germany [43]	CECT	19	tumor > = 2 cm	cTACE	3D	NA	1, AP + PVP	NA	OS
2018, Kim, Korea [44]	CECT	88	BCLC-A/B/C	cTACE	3D	LASSO	12, AP	NA	OS
2020, Meng, China [45]	CECT	162	BCLC-A/B	cTACE	3D	LASSO	6, AP + PVP	internal validation	OS
2020, Peng, China [46]	CECT	798	BCLC-B	NA	2D	CNN	NA, AP	internal and external validation	OR
2021, Guo, China [47]	CECT	94	BCLC-A/B/C	cTACE	2D	LASSO	4, PS	internal validation	OR, OS
2021, Ivanics, Canada [48]	CECT	88	within Milan / Toronto criteria	NA	3D	LASSO, SVM	27, PVP	fivefold cross-validation	Progression dropout in LT list or after LT
2021, Niu, China [49]	CECT	218	BCLC-A/B	cTACE	3D	LASSO	8, AP	external validation	OS
2021, Tipaldi, Italy [50]	CECT	50	BCLC-A/B/C	DEB-TACE	3D	LR	4, PVP	Bootstrap validation	OS
2022, Dai, China [51]	CECT	102	BCLC-B/C	cTACE or DEB-TACE	3D	LASSO	9, PS	internal validation	OS
2022, Liu, China [52]	CECT	70	NA	NA	3D	LASSO	3, NA	tenfold cross-validation	OS
2022, Wang, China [36]	CECT	543	NA	NA	3D	LASSO	25, PVP	internal and external validation	TTP, OS
2023, Fan, China [53]	CECT	92	BCLC-A/B/C	cTACE	3D	adaLASSO	17, AP + PVP	internal validation	Nonviable lesion
2020, Song, China [34]	CEMR	184	BCLC-0/A/B	cTACE	3D	LASSO	14, PVP	internal validation	RFS
2021, Kuang, China [21]	CEMR	153	tumor < = 5 cm, < = 2 lesions	cTACE	3D	mRMR, LASSO	11, AP	external validation	OR
2022, Liu, China [35]	CEMR	140	BCLC-A/B/C	cTACE	3D	mRMR, LASSO	6, T2WI	external validation	OR

NA, not applicable/not available; CECT, contrast-enhanced computed tomography; CEMR, contrast-enhanced magnetic resonance; Gd-EOB-DTPA MR, Gadolinium ethoxybenzyl diethylenetriamine pentaacetic acid-enhanced magnetic resonance; CEUS, contrast-enhanced ultrasound; HCC, hepatocellular carcinoma; BCLC, Barcelona Clinic Liver Cancer; CNLC, China Liver Cancer Staging; TACE, transarterial chemoembolization; cTACE, conventional TACE; DEB-TACE, TACE with drug-eluting beads; mRECIST, modified response evaluation criteria in solid tumors; OR, objective response; OS, overall survival; TTP, time to progression; EVIT, extrahepatic spread or vascular invasion after initial TACE monotherapy; AP, arterial phase; PS, plain scan; PVP, portal vein phase; DP, delayed phase; DL, deep learning; ML, machine learning; CNN, convolutional neural network; LASSO, least absolute shrinkage and selection operator; XGBoost, extreme gradient boosting; LR, logistic regression; mRMR, maximum correlation–minimum redundancy; SVM, support vector machines; RF, random forest; DNN, Deep-Learning Neural Network; LDA, linear discriminant analysis

In terms of imaging modalities, all studies utilized contrast-enhanced examinations. The most frequently investigated imaging modality was contrast-enhanced CT [20, 36–53], followed by contrast-enhanced MR [21, 28–35], with only one article exploring contrast-enhanced ultrasound [54]. Various phases or sequences of contrast-enhanced images were studied, with 18 articles incorporating single-phase/sequence images, while the remaining literature analyzed multi-phase/sequence images. The most commonly utilized phases for radiomics analysis were the arterial phase (AP) [21, 39, 42, 44, 46, 49] and the portal venous phase (PVP) [31, 34, 36, 38, 48, 50]. Regarding MR-based radiomics, T2WI was the most frequently analyzed sequence [29, 32, 35]. Several studies compared radiomics models constructed from images of different phases/sequences, but the conclusions were inconsistent. Some indicated that multi-phase/sequence models were superior [20, 30, 42] in performance, while others demonstrated that single-phase/sequence models were comparable to, or even better than, the multi-phase/sequence models [35, 48]. In addition, comparative studies between different single-phase models suggested that the prognostic value of PVP-based models might be superior to AP-based models [31, 34, 48].

The majority of studies employed 3D volumes of interest (VOIs) for analysis, with only two articles utilizing 2D regions of interest (ROIs) based on the maximum tumor section [46, 47]. Most studies only delineated the tumor regions, while a few explored radiomics features in the peritumor areas [36, 37, 45]. Song et al. suggested that the prognostic value of radiomics models based on tumor regions plus peritumor extensions was not as good as the models considering solely the tumor regions [34]. 28 articles reported the algorithms used for feature selection and model construction, with the majority employing machine learning (ML) algorithms. Five articles adopted deep learning (DL) algorithms in the modeling process [32, 33, 39, 46, 54]. A total of 44 independent datasets (training sets or validation sets) reported both sample sizes of the cohorts and the number of features in the predictive models. The median sample-ize-to-feature-number ratio was 9.30 (p 25–p 75: 5.16–12.43), with only 19 datasets having a value over 10.

### Quality assessment

**Fig. 2:**
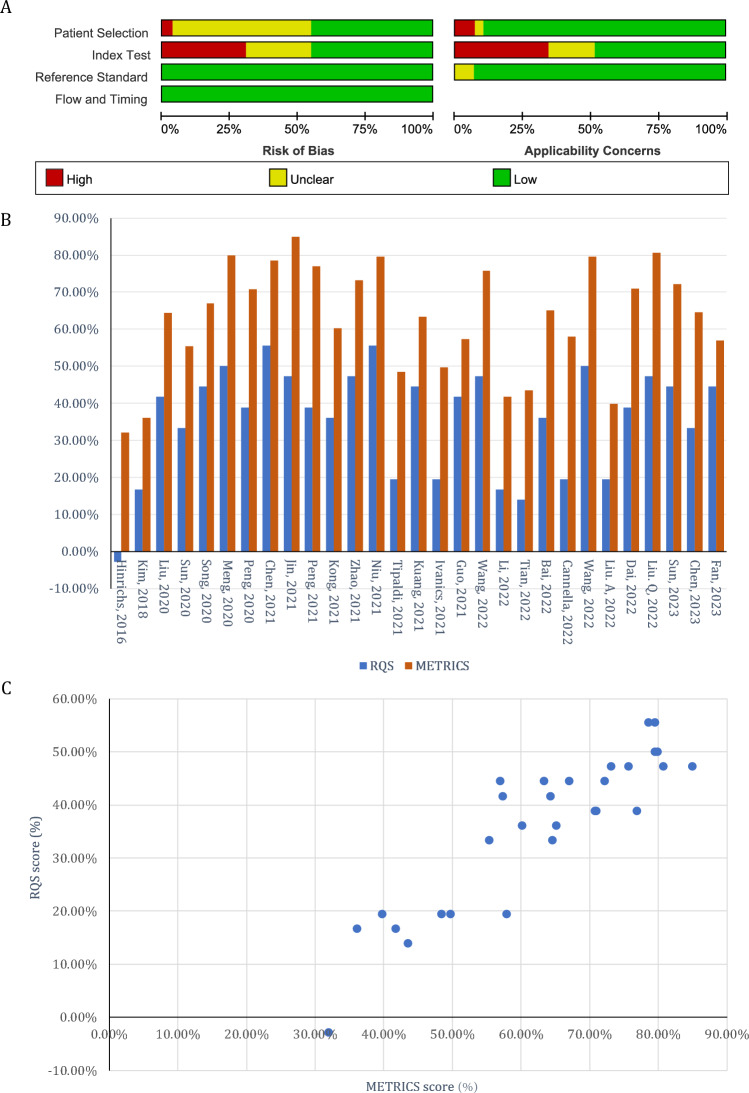
Quality assessment of included articles. **A** The bias risk assessment of included studies utilizing the QUADAS-2 scale. **B** RQS and METRICS scores of included articles. **C** Relationship between METRICS and RQS for each included study, illustrated by scatter plot

A quality assessment was conducted for the included studies. When assessing the risk of bias using QUADAS-2, prevalent biases were identified, originating from unclear case selection procedures (whether all patients were included consecutively), the absence of specified assessment criteria for diagnostic models (cut-off values), and the lack of validation of established models in independent datasets (Fig. 2a, Supplementary Fig. 2A). The latter two factors were also the primary sources of concerns about the studies' applicability.

The summary of study quality according to RQS and METRICS is depicted in Fig. 2b, c, with detailed results presented in Supplementary Fig. 2B and Supplementary Table 5. The overall RQS across all 29 included studies averaged 12.90 ± 5.13 (35.82% ± 14.25%). METRICS scores showed positive correlation with RQS scores (Fig. 2c), averaged 62.98% ± 14.58%. Generally, most studies demonstrated satisfactory quality in terms of reporting imaging strategies, multiple segmentations, integrating non-radiomics clinical variables, assessing the model’s discriminative powers and calibration, model validation, and disclosing radiomics features within the model. Using the METRICS assessment, there were 2, 14, 10 and 3 studies meeting the criteria of “excellent”, “good”, “moderate” and “low”, respectively.

### Study data synthesis (*Meta*-analysis)

#### Pooled predictive performance of radiomics for TACE response

**Fig. 3:**
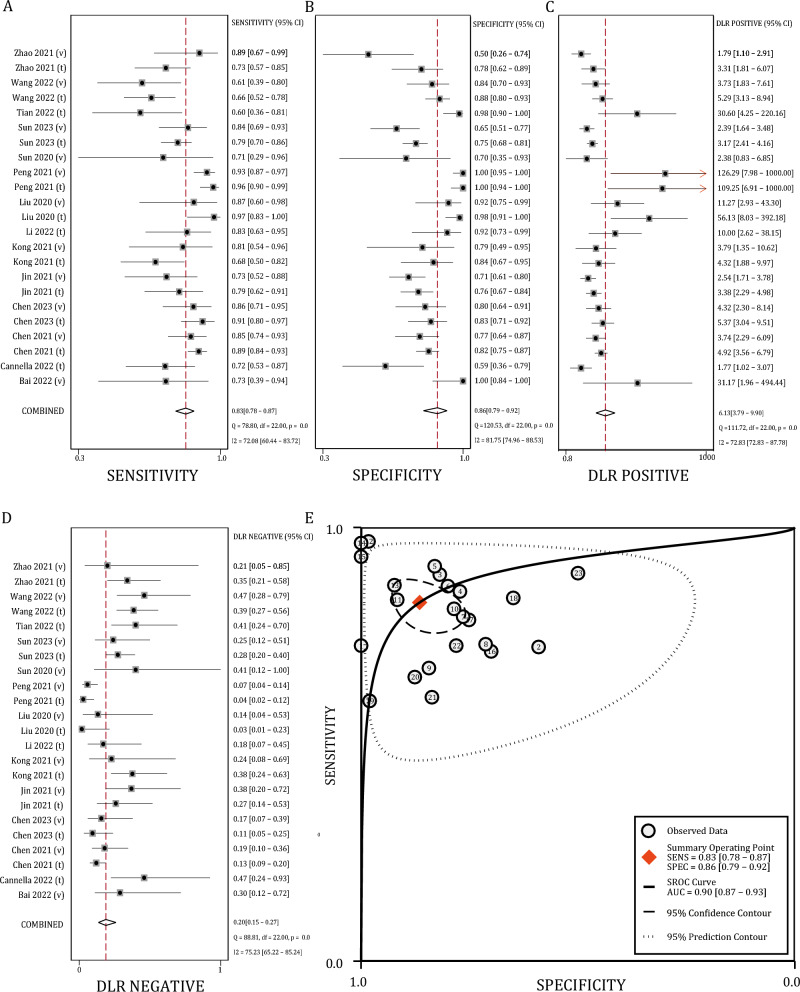
Summarized and pooled performance of radiomics models for predicting TACE response. **A** Pooled sensitivity. **B** Pooled specificity. **C** Pooled positive likelihood ratio. **D** Pooled negative likelihood ratio. **E** The summary receiver operating characteristic curve

**Fig. 4:**
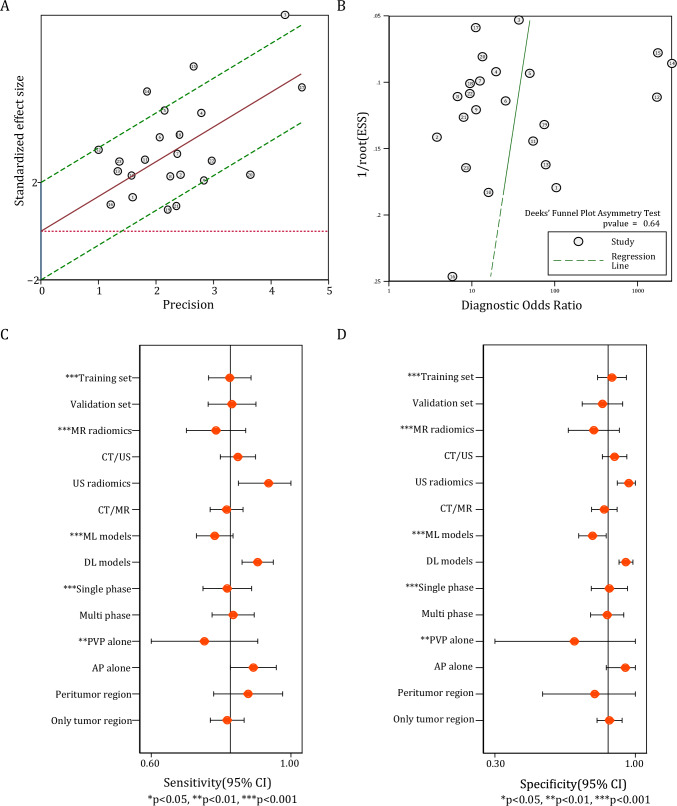
Heterogeneity among included studies. **A** Impact of outliers on the meta-analysis, illustrated by Galbraith plot. **B** No significant publication bias was indicated by Deek’s funnel plot and asymmetry test. **C**–**D** Impact of methodological factors on (**C**) pooled sensitivity and (**D**) specificity of radiomics models, according to meta-regression

A total of 23 independent datasets from 14 studies provided sufficient information to extract a complete 2 × 2 contingency table and thus entered the meta-analysis. The included datasets comprised 12 training datasets (with or without resampling validation), including 1628 patients, and 11 independent validation datasets (internal or external validation), comprising 815 patients. In all of these datasets, the predictive endpoint of radiomics models was the tumor response after TACE, defined by objective response (OR) according to the RECIST [14] or mRECIST [15] criteria in the majority of the studies. All datasets included in the meta-analysis and their key methodological information are outlined in Table 2. For the total of 23 datasets, a synthetic analysis of predictive performance was conducted on 2443 subjects (Fig. 3a–e, Supplementary Table 6). The pooled sensitivity was 0.83 (95% CI: 0.78–0.87) (Fig. 3a), specificity was 0.86 (95% CI: 0.79–0.92) (Fig. 3b), and the pooled PLR and NLR were 6.13 (95% CI: 3.79–9.90) (Fig. 3c) and 0.20 (95% CI: 0.15–0.27) (Fig. 3d), respectively. The AUC of the sROC was 0.90 (95% CI: 0.87–0.93) (Fig. 3e). Heterogeneity tests revealed *I*^2^ exceeding 70% for sensitivity, specificity, PLR, and NLR, with *Q*-test *P*-values below 0.01, indicating significant heterogeneity (Supplementary Table 6). Further Galbraith plot suggested a limited and symmetrical impact of outliers on the result of the meta-analysis (Fig. 4a).

**Table 2:** Major results and methodological features of the studies in meta-analysis

Author year	TP	FP	FN	TN	Dataset	Modality	Algorithm	Phase	AP/ PVP	Peritumor
Bai 2022 (v)	8	0	3	21	validation	CT	ML	Multi		No
Cannella 2022 (t)	21	9	8	13	training	MR	ML	Single	PVP	No
Chen 2021 (t)	164	31	20	140	training	CT	ML	Multi		Yes
Chen 2021 (v)	52	13	9	44	validation	CT	ML	Multi		Yes
Chen 2023 (t)	51	10	5	49	training	MR	DL	Multi		No
Chen 2023 (v)	32	8	5	32	validation	MR	DL	Multi		No
Jin 2021 (t)	27	24	7	78	training	CT	ML	Single	PVP	No
Jin 2021 (v)	19	27	7	67	validation	CT	ML	Single	PVP	No
Kong 2021 (t)	25	5	12	27	training	MR	ML	Single		No
Kong 2021 (v)	13	3	3	11	validation	MR	ML	Single		No
Li 2022 (t)	20	2	4	22	training	CT	ML	Multi		No
Liu 2020 (t)	30	1	1	57	training	US	DL	Multi		No
Liu 2020 (v)	13	2	2	24	validation	US	DL	Multi		No
Peng 2021 (t)	80	0	3	56	training	CT	DL	Single	AP	No
Peng 2021 (v)	97	0	7	67	validation	CT	DL	Single	AP	No
Sun 2020 (v)	5	3	2	7	validation	MR	ML	Multi		No
Sun 2023 (t)	96	44	26	133	training	CT	ML	Single	AP	No
Sun 2023 (v)	36	20	7	37	validation	CT	ML	Single	AP	No
Tian 2022 (t)	12	1	8	50	training	MR	DL	Single		No
Wang 2022 (t)	38	14	20	99	training	CT	ML	Multi		No
Wang 2022 (v)	14	8	9	41	validation	CT	ML	Multi		No
Zhao 2021 (t)	32	9	12	32	training	MR	ML	Multi		No
Zhao 2021 (v)	17	9	2	9	validation	MR	ML	Multi		No

(t), training dataset; (v), validation dataset; TP, true positive; FP, false positive; FN, false negative; TN, true negative; CT, contrast-enhanced computed tomography; MR, magnetic resonance; AP, arterial phase; PVP, portal vein phase; DL, deep learning; ML, machine learning

#### Origin of heterogeneity and subgroup analysis

Meta-regression analysis was performed based on the key methodological parameters, which incorporated six variables: imaging modalities (CT/MR/US), training or validation datasets, modeling algorithms (ML/DL), imaging phases (single-phase/multi-phase), single imaging phase (AP/PVP), and the inclusion of peritumoral features (Yes/No). In relation to pooled sensitivity, noteworthy inter-subgroup variances were identified between training sets and validation sets, MR studies and other studies, ML models and DL models, single-phase models and multi-phase models, as well as AP models and PVP models (Fig. 4c, Supplementary Table 7). Concerning specificity, significant disparities were discerned between MR studies and other studies, and between ML models and DL models (Fig. 4d, Supplementary Table 7).

Subsequent subgroup analyses were conducted for variables deemed significant in the meta-regression. Due to the limited studies reporting single-phase AP models and PVP models, subgroup analyses were executed based on the remaining factors, and pooled sensitivity, specificity, and heterogeneity within each subgroup was calculated (Table 3). Inter-study heterogeneity was significantly lower among the independent validation datasets compared to the training datasets, while comparable model performance was reported (*I*^2^: 51.32% vs. 82.11%, Supplementary Fig. 3). In the 11 validation datasets, further subgrouping was based on imaging modalities, imaging phases and modeling algorithms. Inter-study homogeneity with *I*^2^ < 50% was observed in the MR subgroup, ML subgroup, and the multi-phase model subgroup (Supplementary Fig. 4–6). Furthermore, the five studies combining CT and ML also showed satisfactory homogeneity (*I*^2^: 45.07, Supplementary Fig. 7).

**Table 3:** The pooled results from subgroup analysis

Subgroups			No. of datasets	Sensitivity (95%CI)	*I*^*2*^% (P value)	Specificity (95%CI)	*I*^*2*^% (P value)
Datasets	Training		12	0.83 (0.74–0.89)	82.11 (0.00)	0.88 (0.79–0.94)	83.43 (0.00)
	Validation		11	0.83 (0.77–0.88)	51.32 (0.02)	0.84 (0.71–0.92)	80.54 (0.00)
Validation Datasets	Modality	CT	6	0.83 (0.72–0.89)	73.45 (0.00)	0.90 (0.64–0.98)	88.65 (0.00)
		MR	4	0.85 (0.75–0.91)	0.00 (0.67) *	0.71 (0.57–0.82)	49.19 (0.12) *
	Algorithm	DL	3	NA (limited data)			
		ML	8	0.79 (0.71–0.85)	22.88 (0.25) *	0.75 (0.66–0.82)	57.08 (0.02)
	Phase	Single	4	0.85 (0.75–0.92)	65.38 (0.00)	0.87 (0.52–0.98)	90.91 (0.00)
		Multi	7	0.81 (0.72–0.88)	38.59 (0.13) *	0.81 (0.71–0.88)	65.32 (0.01)
	MR + ML		3	NA (limited data)			
	CT + ML		5	0.77 (0.67–0.85)	45.07 (0.12) *	0.77 (0.68–0.85)	63.27 (0.03)

*refer to synthesized results with I^2^ < 50% and P > 0.1

#### Evaluation of publication *bias* and clinical interpretation

**Fig. 5:**
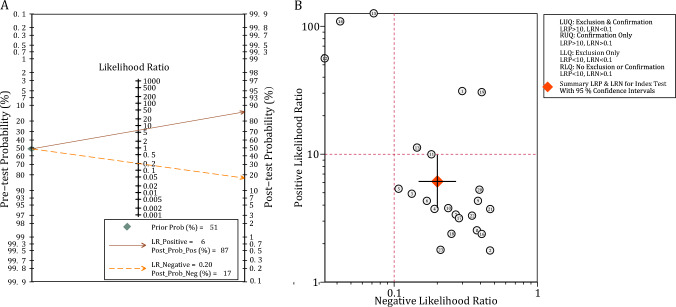
Clinical utility of the radiomics models. **A** Fagan plot illustrating the posterior probabilities of the radiomics models. **B** Likelihood ratio matrix illustrating the informative value of each individual radiomics model

The funnel plot disclosed no significant evidence of publication bias among the studies included in the analysis (*P* = 0.64, see Fig. 4b). Of the 20 datasets extracted from 11 studies that reported ORR following TACE, the weighted average ORR was determined to be 51.24% (Supplementary Table 8). Using this average ORR as the pre-test probability, the posterior probabilities of achieving an objective response were calculated to be 87% and 17% when the radiomics models yielded positive and negative predictions, respectively (Fig. 5a). The likelihood ratio matrix illustrated that the majority of studies failed to attain optimal PLR and NLR, with only three records falling within the upper left quadrant, indicative of high predictive informativeness (Fig. 5b).

## Discussion

This systematic review encompassed a total of 29 primary studies, involving 5483 patients, with 14 articles integrated into the meta-analysis, constituting 2691 patients. The studies manifested noteworthy regional disparities, as 25 studies (86.2%) emanated from East Asia, of which 24 hailed from China (82.8%), including a total of 5187 Chinese patients (2640 Chinese patients in the meta-analysis). This observation aligns with the high incidence of HCC in East Asia [1] and the prevalent utilization of TACE within this region [6]. Additionally, this regional concentration determined a high proportion of viral-hepatitis-related HCC cases in this review (Supplementary Table 4). We further performed an exploratory bibliometric analysis by searching the Web of Science core database. The findings revealed that 42.3% of publications within the purview of "radiomics" originated from China. Similarly, Chinese researchers contributed 30.2% and 31.4% of the studies on "HCC" and "TACE", respectively, establishing China as the foremost source of studies in these domains. From the perspective of practical implication, radiomics tools may confer cost advantages and greater convenience compared to molecular-pathological tests, such as next-generation sequencing, especially in middle-income countries like China. Nevertheless, this does not diminish the applicational potential these studies in the context of global HCC management and precision medicine.

Among the included articles, mid-term HCC patients, predominantly BCLC-B stage patients, constituted the predominant cohorts, aligning with the prevailing indications for TACE [5, 13]. The majority of the studies delved into the predictive value of cross-sectional imaging, encompassing CT and MR, both acknowledged as diagnostic modalities for HCC [65]. This alignment with the traditional clinical and radiological practice might set the basis of convenient application of these radiomic models. Since 2018, a discernible upswing in the number of studies in this field has been noted, and the studies showed considerable consistency in research patterns, indicating a trend of maturation and standardization of radiomics research [59, 60].

To our knowledge, this represents the inaugural systematic review and meta-analysis delving into the predictive efficacy of radiomics in determining the prognosis of ACE. Previous investigations endeavored to harness pre-treatment conventional imaging features for predicting TACE outcomes. Parameters such as tumor size, location, enhancement patterns, enhancement heterogeneity, and intraprocedural digital subtraction angiography (DSA) characteristics were explored [66, 67]. Additionally, established liver imaging assessment tools, like the liver imaging reporting and data system (LI-RADS), were also employed for this purpose [68]. Though these traditional imaging features had demonstrated some correlations with TACE response, they were not specifically tailored tools for this purpose, resulting in imprecise prediction and limited clinical value. Consequently, radiomics methodologies emerge as a promising avenue in this context. Radiomics has demonstrated substantial potential across various oncological domains since its inception. Previous systematic reviews and meta-analyses have consolidated evidence of its efficacy in diverse areas, such as predicting microvascular invasion (MVI) in HCC [69, 70], post-radiotherapy survival in non-small cell lung cancer (NSCLC) [71], and differential diagnosis and prognosis prediction for renal cell carcinoma (RCC) [72]. Diverging from preceding meta-analyses, our study placed particular emphasis on the generalizability of radiomics models. We independently analyzed outcomes from both the datasets utilized for model training and the separate datasets designated for validation. The combined results from validation sets did not disclose insufficient model performance compared to training sets. This finding lays the groundwork for future endeavors in validating and applying these radiomics models. Of note, utilization of public datasets for model testing is a practical way to enhance the generalizability of radiomic models. In terms of the specific tasks explored in this meta-analysis, the HCC-TACE-SEG dataset from The Cancer Imaging Archive (TCIA) might provide a proper external validation cohort [73], which is worth exploiting in future studies.

In this systematic review, we utilized QUADAS-2 [58], RQS [59] and METRICS [60] to appraise the quality of radiomics studies. In general, the included studies showed moderate to good quality. In 2020, Ursprung et al. summarized the role of radiomics in RCC, reporting an average RQS of 3.41 ± 4.43 for 57 articles [72]. In contrast, this systematic review, comprising 29 studies, reported a significantly higher average RQS of 12.90 ± 5.13, reflecting the advancements in this field in recent years. Further analysis of the 16 scoring categories in RQS revealed the most pronounced disparity in the "validation" category, while the lowest scores were noted in three domains: "detect and discuss biological correlates," "prospective study registered in a trial database," and "cost-effectiveness analysis." This discrepancy may be partly attributed to our specific inclusion criteria, where prospective studies could be constrained by the intricacies of treatment indications [12], and the joint analysis with other biomarkers might be impeded by the absence of reliable predictive biomarkers for TACE efficacy. However, it is noteworthy that integrating cost-effectiveness analysis could play a pivotal role in the subsequent validation and application of radiomics models, particularly in evaluating methodological factors such as the workforce and time required for image segmentation, additional resources for multi-phase image segmentation, and net benefits compared to traditional imaging examinations. In addition, we evaluated METRICS scores of the studies, which is a novel assessment tool that is more detailed and balanced compared to RQS. For most included studies, further improvement could be made in handling clinical confounding factors, appropriate use of machine learning algorithms, appropriate choice of imaging phases, independent validation of the results, as well as the transparency and reproducibility of scientific data. Hence, we advocate for future radiomics research to incorporate these considerations in their analysis and reports.

The meta-analysis involving all the included radiomics models indicated pooled sensitivity and specificity surpassing 0.80. As these models were developed in highly bespoke manners within retrospective cohorts, lacking consistent cut-off values across different models, we employed a summary receiver operating characteristic to assess the general prognostic performance, which unveiled an AUC of 0.90, signifying robust discriminative capacity for post-TACE prognosis. As indicative parameters for clinical application, the pooled PLR and NLR were 6.13 and 0.20, respectively, with only three studies meeting the criteria of PLR > 10 and NLR < 0.1. Overall, the current radiomics models demonstrated moderate clinical informative value. Furthermore, with prior probability taken into consideration, the calculated post-test probabilities of objective response were 87% and 17% when positive and negative results were given by the radiomics models, respectively. Accordingly, radiomics tools could forecast totally different outcomes after TACE, and thus harbor significant potential in informing therapeutic decisions of TACE.

Significant heterogeneity surfaced among the incorporated studies, a revelation consistent with prior meta-analyses in the radiomics field [69–72], aligned with our expectations. Given the retrospective design of all studies, heterogeneity was inevitable [70]. Although similar and standard research workflows were adopted by most of the included studies, numerous methodological variables in the whole analyzing process, from imaging data to the final predictive model, could contribute to substantial disparities among studies, which is an inherent limitation of highly customized models. Nevertheless, the combined statistical results of the current models are still of considerable value. In this study, we utilized the Galbraith plot to evaluate the impact of heterogeneity on the meta-analysis results, uncovering few extreme outliers and a generally symmetric impact. Furthermore, Deek’s funnel plot and asymmetry tests were utilized, suggesting no apparent publication bias.

Through meta-regression, we scrutinized the origins of heterogeneity, we observed that several crucial methodological factors including imaging modalities, modeling algorithms, and validation or training datasets could potentially be major contributors to inter-study heterogeneity. In subgroup analyses, results from testing sets among studies that adopted the same imaging method (MR), imaging phases (multi-phase imaging data), or the same type of modeling (ML-based modeling) showed better homogeneity. And the synthesized results in subgroups did not show serious deviation from the combined results of all studies. Taken together, we believe that radiomics tools have great potential for predicting treatment outcome of TACE, from both the research and clinical application perspectives.

We analyzed the performance of different models only in the validation datasets (Table 3), CT-based radiomics models demonstrated comparable pooled sensitivity and superior specificity compared to MR models. Considering the lower time cost and expense of CT compared to MR, CT-based radiomics models may hold an advantage in practical clinical application. Interestingly, models using single-phase/sequence imaging data did not show insufficient performance compared to multi-phase/sequence models in combined sensitivity and specificity. A few studies directly compared the predictive value between uniparametric and multi-parametric models [20, 30, 35, 42, 48], and there was no consistent evidence supporting joint analysis of multiple imaging phases or sequences. As pointed out by the METRICS guideline [60], multi-parametric analysis may unnecessarily increase the data dimensionality and risk of overfitting. Therefore, future studies should try to use simple radiographic data or prove the added value of multi-parametric models. Notably, while many studies considered multiple phases, very few conducted voxel-to-voxel image matching across different phases [43, 49], with no study analyzing the evolution of feature values across different time points. Future research employing image alignment and feature matching to analyze the time evolution of features might expand the role of delta-radiomics [59] and fully leverage the potential of multi-phase imaging.

The choice of modeling algorithms influences the model's performance in a decisive manner [74, 75]. In our subgroup analysis, ML-based models demonstrated inferior sensitivity and specificity compared to DL models. In addition, two studies directly compared DL and ML models and indicated the superior predictive power of DL models [33, 54]. However, it is noteworthy that the transparency of details in DL models was limited in the included studies, potentially restricting the reproducibility of their results. Generally, deep learning demonstrated significant potential for radiomics modeling, albeit its current limited use in studies [32, 33, 39, 46, 54]. There is presently no consensus on the necessity of including peritumoral features. Our meta-regression and the direct comparison by Song et al. [34] did not reveal significant benefit of considering peritumoral regions. Nevertheless, in other tasks, such as predicting MVI [76] and postoperative recurrence [77] in HCC, analyzing both tumor and peritumoral regions proved to be more effective. Hence, whether incorporating peritumoral features improves model performance might be task-specific.

Finally, while our meta-analysis suggested potential advantages of some methods, there have been no universally recognized optimal choices for these methodological options, and further validations are imperative. The ongoing exploration and standardization of radiomics research methods will undoubtedly lead to more ideal predictive models, guiding decision-making and management for HCC patients undergoing TACE treatment.

## Limitations and future perspectives

This study presents several limitations. Firstly, all included studies are retrospective in design. Despite conducting meta-regression and subgroup analyses, the sources of heterogeneity could not be fully elucidated. Therefore, caution is advised when directly applying the results of these studies due to potential concerns about generalizability. This limitation underscores the necessity for further methodological exploration and additional radiomics research to address these gaps. Secondly, not all included datasets met the ideal sample size criterion of ten times the number of model features, emphasizing the need for future studies to establish larger clinical cohorts or reduce the number of model features.

## Conclusion

Radiomics models demonstrated good accuracy and promising clinical utility in predicting the prognosis of initial TACEtreatment. Methodological factors such as imaging modalities, phases of imaging, and modeling algorithms are significant sources of literature heterogeneity.

### Supplementary Information

Below is the link to the electronic supplementary material.

Supplementary file1 (XLSX 218 KB)

Supplementary file2 (PDF 3140 KB)

## Data Availability

All articles included in this review are available from PubMed, Web of Science, and Embase. All data generated during the analysis in this study are included in the article and supplementary materials.

## Abbreviations

TACE: Transarterial chemoembolization
cTACE: Conventional TACE
DEB-TACE: Drug-eluting bead TACE
HCC: Hepatocellular carcinoma
MVI: Microvascular invasion
NSCLC: Non-small cell lung cancer
RCC: Renal cell carcinoma
BCLC: Barcelona clinic liver cancer
CNLC: China liver cancer staging
RECIST: Response evaluation criteria in solid tumors
mRECIST: Modified response evaluation criteria in solid tumors
OR: Objective response
ORR: Objective response rates
OS: Overall survival
MR: Magnetic resonance
Gd-EOB-DTPA: Gadolinium ethoxybenzyl diethylenetriamine pentaacetic acid
CT: Computed tomography
US: Ultrasound
DSA: Digital subtraction angiography
LI-RADS: Liver imaging reporting and data system
AP: Arterial phase
PVP: Portal venous phase
VOIs: Volumes of interest
ROIs: Regions of interest
PRISMA-DTA: Preferred reporting items for systematic review and meta-analysis of diagnostic test accuracy studies
PICO: Participants, intervention, control, outcome
QUADAS-2: Quality assessment of diagnostic accuracy studies 2
RQS: Radiomics quality score
METRICS: METhodological RadiomICs Score
PLR: Positive likelihood ratio
NLR: Negative likelihood ratio
sROC: Summary receiver operating characteristic curve
AUC: Area under curve
95% CI: 95% confidence intervals
TP: True positive
TN: True negative
FP: False positive
FN: False negative
DL: Deep learning
ML: Machine learning
