# Supplementary material for: Radiomics as a tool for prognostic prediction in transarterial chemoembolization for hepatocellular carcinoma: a systematic review and meta-analysis
Source: Radiol Med. 2024 Jul 26;129(8):1099–117. doi: 10.1007/s11547-024-01840-9 (PMC11322429; doi:10.1007/s11547-024-01840-9)
Supplement: Supplementary file 2 — Supplementary file2 (PDF 3140 KB) [file 11547_2024_1840_MOESM2_ESM.pdf]

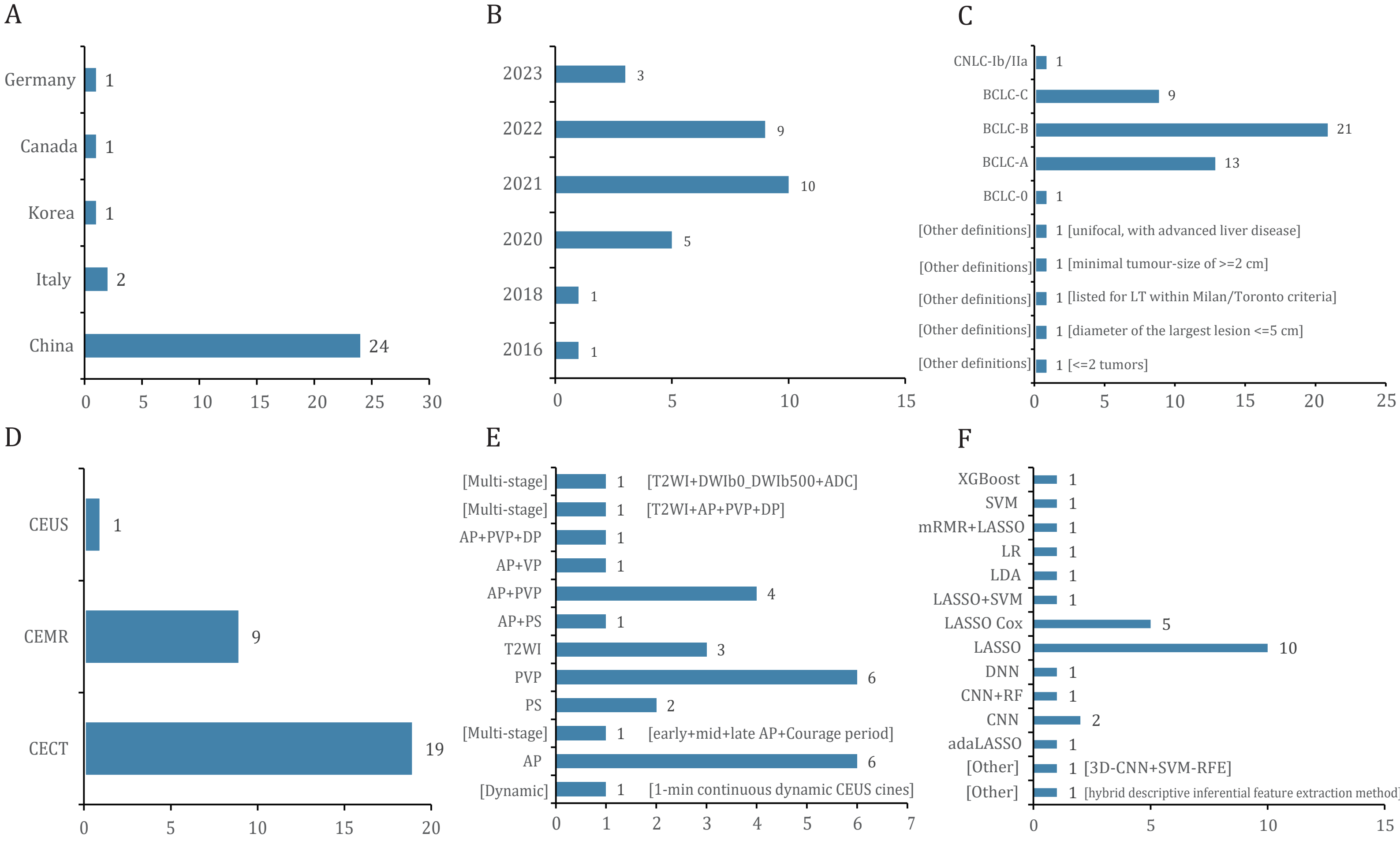

Supplementary Figure 1. Major Characteristics of Included Studies. (A-B) The number of published studies across (A) different countries and (B) years. (C) Frequency distribution of various tumor stages investigated in the included research, with reference to Barcelona Clinic Liver Cancer (BCLC) and China Liver Cancer Staging (CNLC). (D) Number of studies employing distinct imaging modalities, including contrast-enhanced computed tomography (CECT), contrast-enhanced magnetic resonance (CEMR), and contrast-enhanced ultrasound (CEUS). (E) Number of studies analyzing diverse phases/ sequences of medical images, such as arterial phase (AP), plain scan (PS), and portal vein phase (PVP). (F) Number of studies utilizing different modelling algorithms, including convolutional neural network (CNN), least absolute shrinkage and selection operator (LASSO), extreme gradient boosting (XGBoost), logistic regression (LR), maximum correlation–minimum redundancy (mRMR), support vector machines (SVM), random forest (RF), deep-learning neural network (DNN), and linear discriminant analysis (LDA).

A

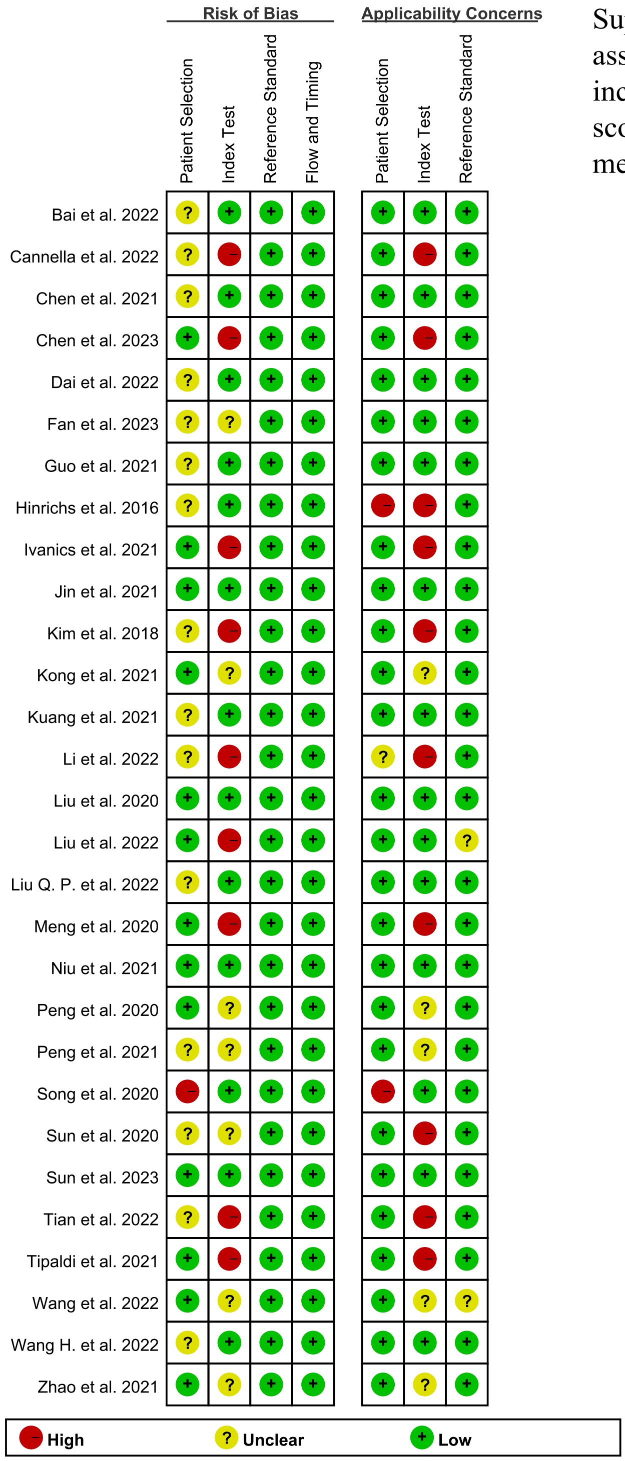

Supplementary Figure 2. (A) Details of QUADAS-2 quality assessment for included studies. (B) Details of RQS assessment for included studies, with minimum and maximum scores for each scoring item across all studies represented by black line segments and mean ± standard deviation depicted by boxes.

B

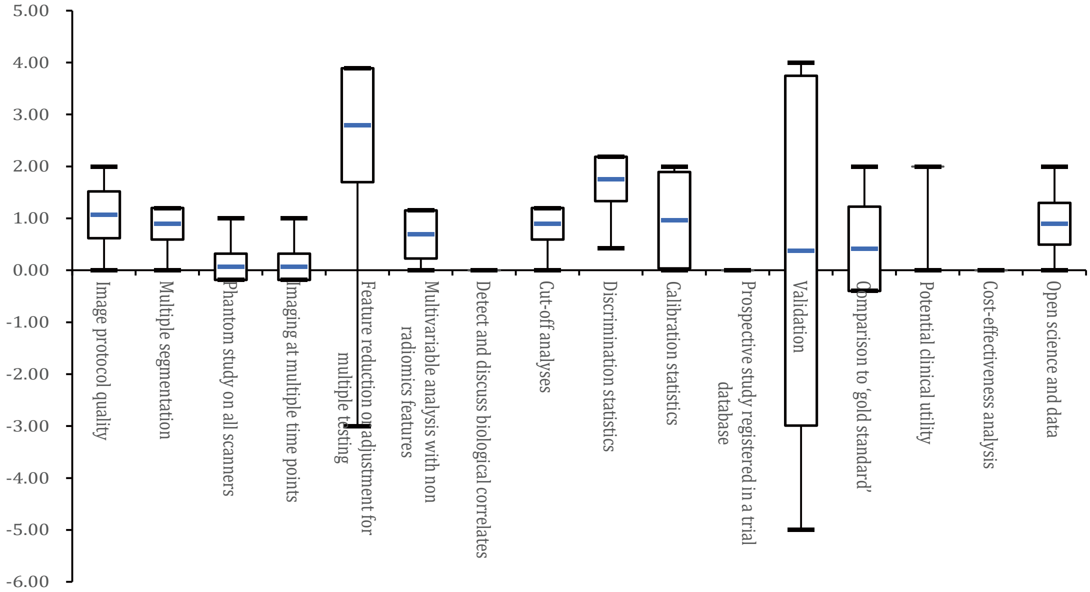

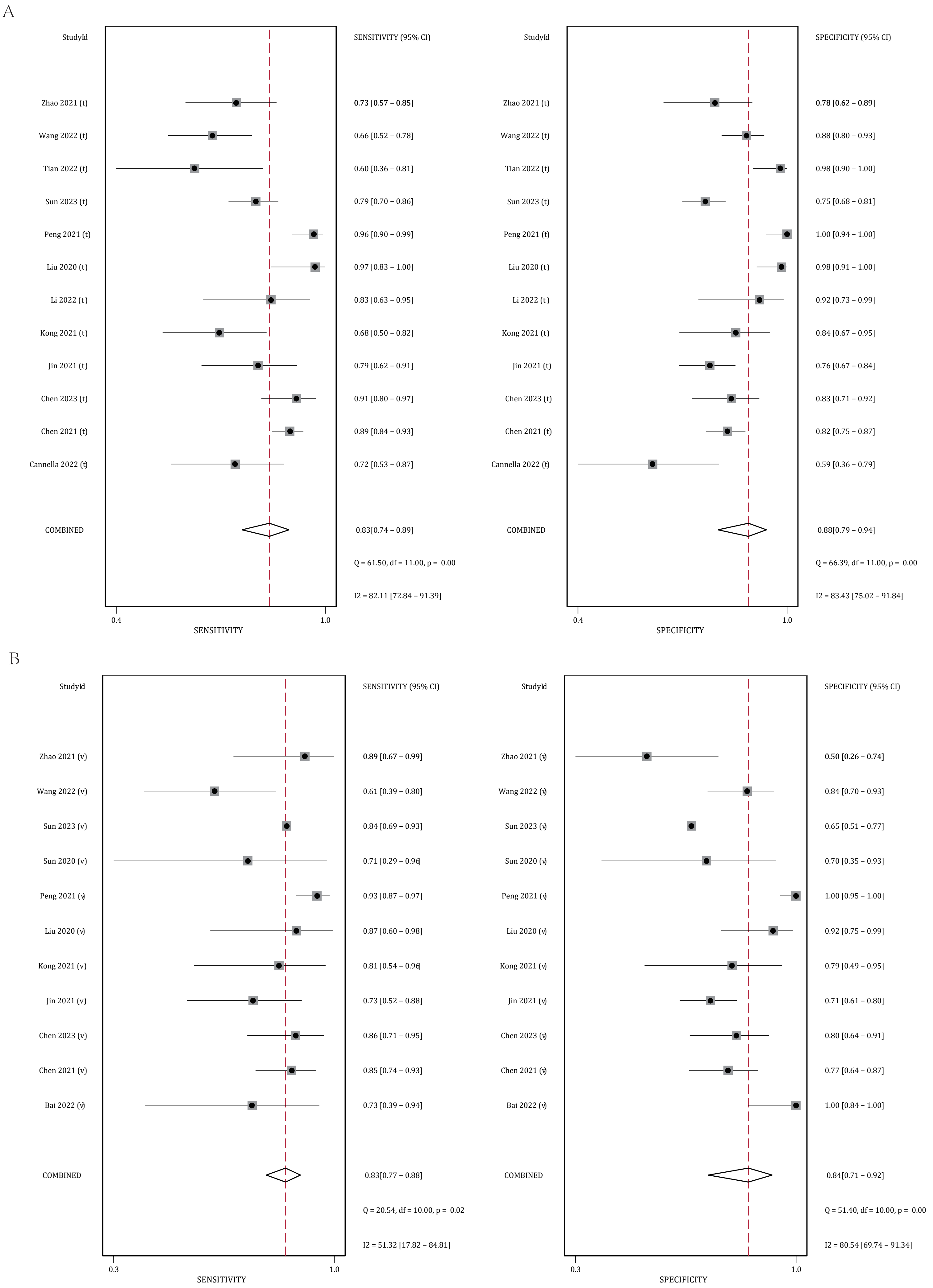

Supplementary Figure 3. Subgroup analysis of models in (A) training sets and (B) validation sets.

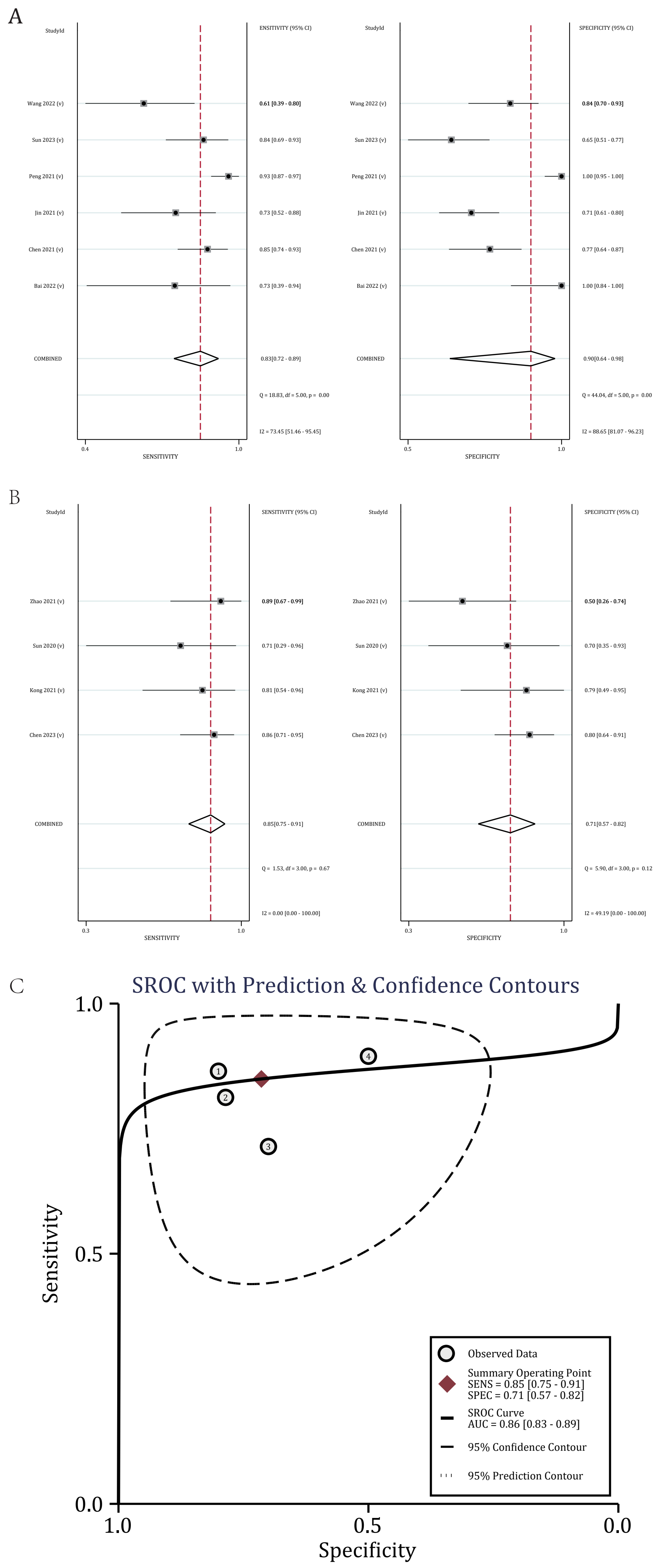

Supplementary Figure 4. Subgroup analysis of models based on (A) CT and (B) MR in validation datasets and (C) the summary ROC in the subgroup MR.

A

B

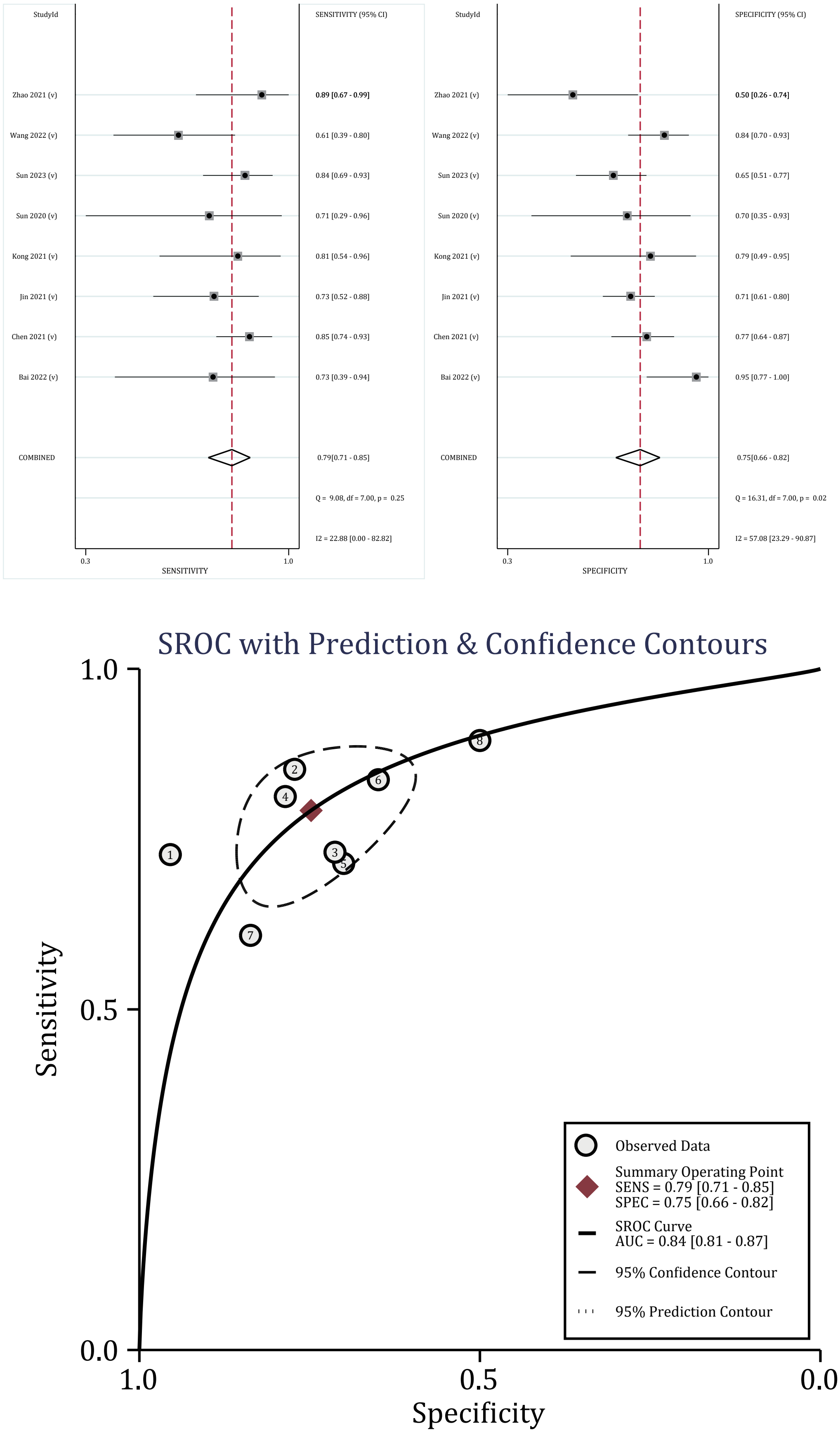

Supplementary Figure 5. Subgroup analysis of machine-learning-based models in (A) validation datasets and (B) the summary ROC in this subgroup.

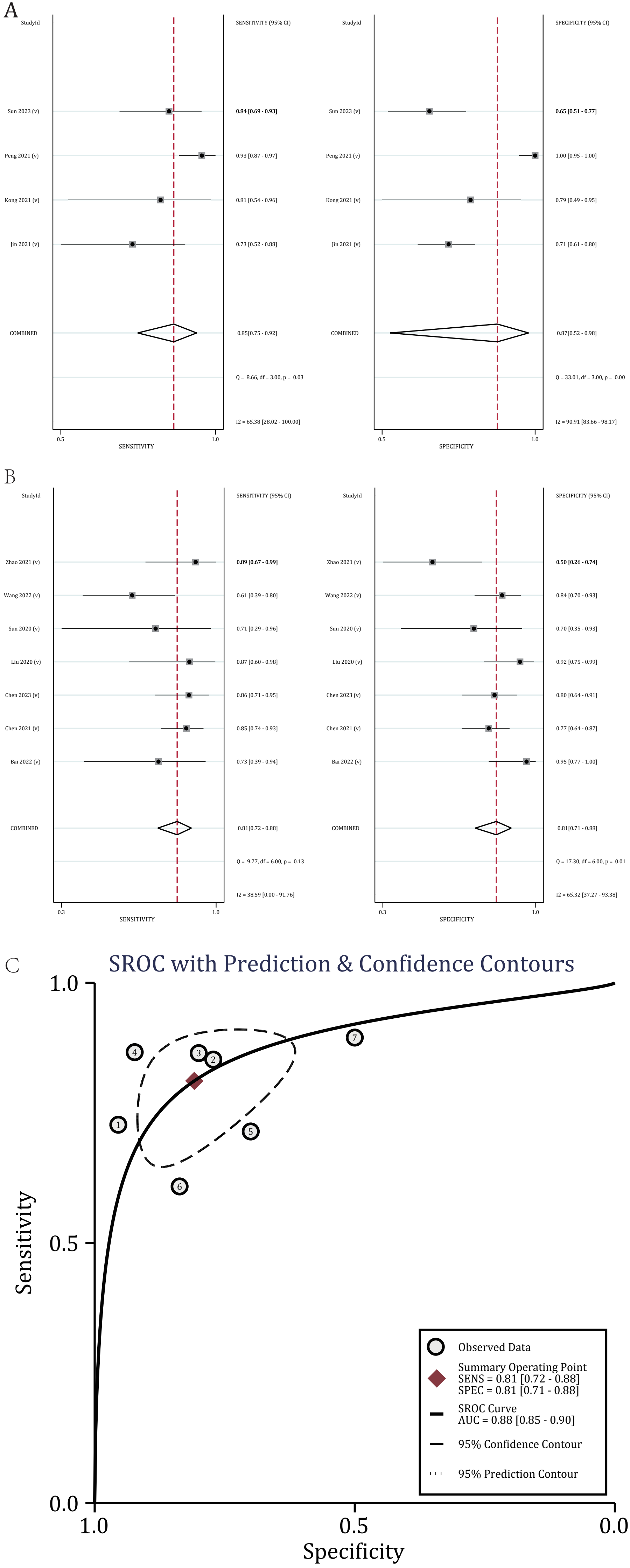

Supplementary Figure 6. Subgroup analysis of models based on (A) single-phase and (B) multi-phase medical images in validation datasets and (C) the summary ROC in the latter subgroup.

A

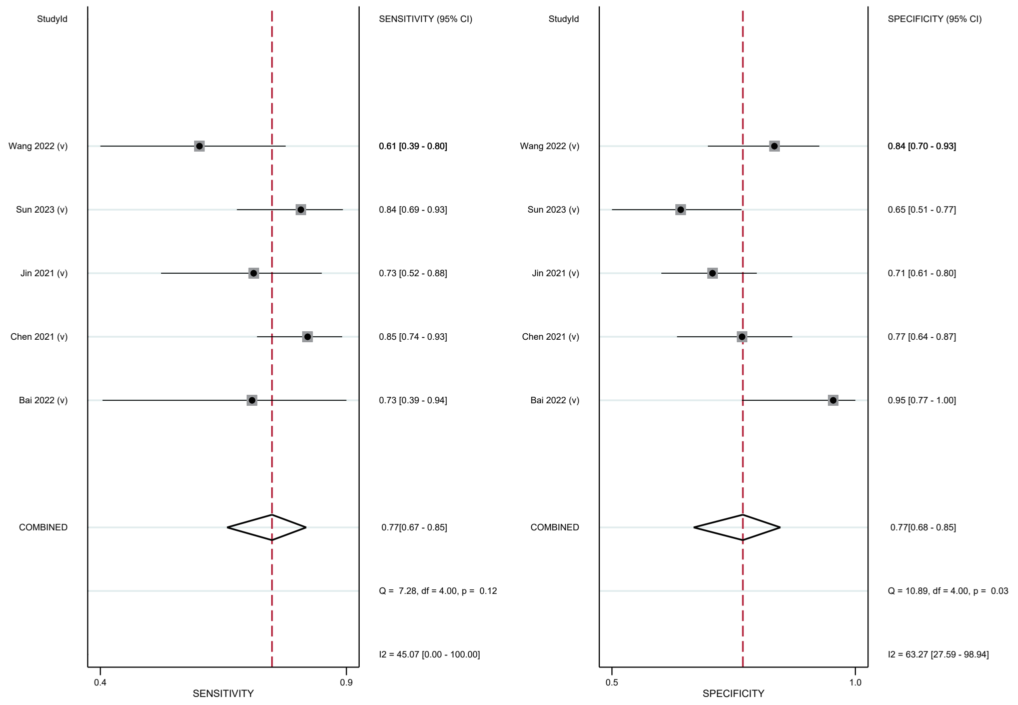

B

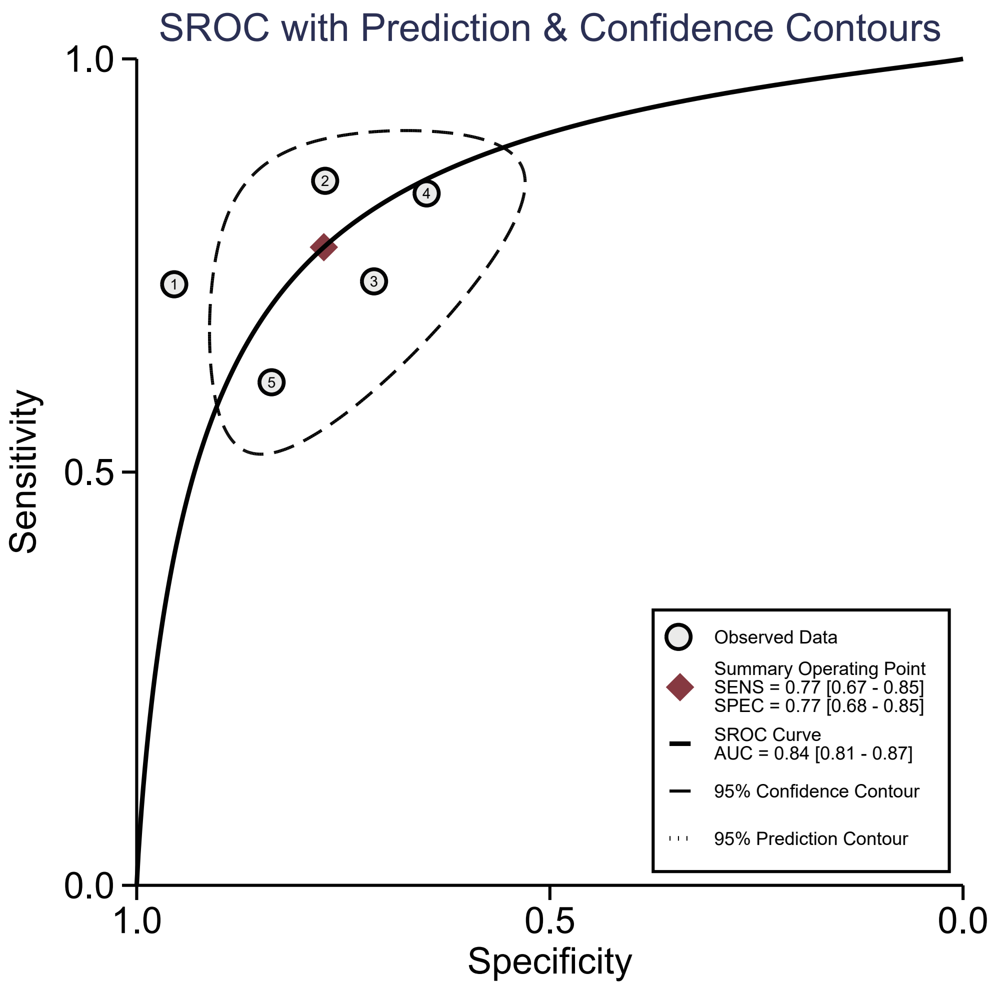

Supplementary Figure 7. (A) Subgroup analysis of models based on CT and machine learning in validation datasets. (B) The summary ROC in this subgroup.
